# The evolution logic and optimisation strategies of China’s emergency management system from the perspective of Punctuated Equilibrium Theory (1949–2023)

**DOI:** 10.3389/fpubh.2026.1802526

**Published:** 2026-04-21

**Authors:** Tian Lai

**Affiliations:** Wuhan University, Wuhan, China

**Keywords:** emergency management, transmutation logic, focal events, Punctuated Equilibrium Theory (PET), public policy

## Abstract

The emergency management system constitutes the core of national emergency preparedness. Analysed through the lens of Punctuated Equilibrium Theory (PET), this study proposes a dual-driver model that integrates both exogenous focal events and endogenous ideological shifts—an amendment extending PET to account for top-down, state-led punctuations in China’s governance context. Since 1949, the system has followed a pattern of gradual equilibrium, policy punctuation, and new equilibrium reconstruction. Four equilibrium periods are identified: disaster-specific management (1949–2002); “One Plan, Three Systems” integrated management (2003–2012); holistic national security outlook (2013–2018); and “Big Security – Big Emergency” integrated governance (2019–present). Three major punctuations and one institutional restructuring are traced. Key characteristics include policy image transformation from single-hazard response to integrated governance, policy venue expansion from unitary centralised management to multi-party collaborative governance, and the increasing salience of focal events. Future optimisation should focus on expanding the policy venue, enhancing scientific policy design, and fostering bidirectional empowerment between top-level design and grassroots momentum.

## 1 Introduction

A review of pertinent literature reveals that academic research on China’s existing emergency management primarily focuses, from the perspective of crisis types, on specific natural disasters (1, 2), mass emergencies (3), food safety (4), and online public opinion (5). From the perspective of the emergency response stage, it encompasses aspects such as risk and vulnerability assessment (6), infrastructure construction and enhancement (7), emergency response (8), emergency resource reserve (9) and allocation (10, 11), interdepartmental cooperation (12) and emergency coordination (13), crisis learning (14), and digital governance (15). These studies provide a robust foundation for understanding specific facets and operational aspects of China’s emergency management system. However, they largely focus on concrete issues at the ‘policy subsystem’ level and rarely engage with theoretical explanations of macro-level policy change.

Concurrently, international scholarship on theories of policy change, particularly Punctuated Equilibrium Theory (PET), has matured and increasingly focused on its applicability across diverse political contexts. Several scholars have endeavoured to apply PET to non-Western, non-democratic settings to test its generalisability. For instance, van den Dool (16) systematically assessed the application of mainstream policy process frameworks—including the Advocacy Coalition Framework, Multiple Streams Framework, and PET—in 39 autocracies, finding that while these theories help identify key institutions, actors, beliefs, and events influencing policy processes, their mechanisms of operation differ significantly from those in democracies. Empirical studies on China (and Hong Kong) by Chan (17) and Lam (18) further reveal that authoritarian regimes, due to their information disadvantages and suppression of opposing voices, may experience more intense policy punctuations than democracies, with the volatility of policy agendas influenced by the degree of power centralisation. These comparative studies offer crucial theoretical benchmarks for understanding the peculiarities of China’s policy transformations.

Furthermore, international scholars have delved deeply into the core concepts, methodologies, and application of PET at the supranational governance level. Kuhlmann (19), through a systematic review of core PET literature, points out that while PET research has greatly enriched our understanding of policy change, conceptual selectivity is common in its application, calling for more rigorous theory testing and knowledge accumulation. Princen (20) introduced PET into the analysis of European Union (EU) policy processes, arguing that the theory, due to its emphasis on attention allocation and the interplay of institutional and ideational factors, is well-suited to handle the complexity of EU multi-level governance—a perspective that offers insights for understanding central-local interactions in China’s emergency management. Methodologically, Kaplaner (21) proposed using the Gini coefficient instead of kurtosis to measure the intensity of policy punctuations, providing a new tool for more precisely characterising patterns of policy change. Moreover, numerous single or comparative case studies, such as analyses of emergency coordination in China (12, 13, 22), the evolution of US energy policy (23), Belgian digital policy (24), and Danish local budgeting (25), all corroborate the explanatory power of the PET framework from different angles, demonstrating the complex interplay of core variables like policy image, policy venue, and focusing events across different institutional contexts.

Although some scholars have endeavoured to review the history of emergency management development in China from a more macroscopic perspective (26–28), extant research still exhibits three principal problems: Firstly, existing research seldom employs established academic theory (such as PET) to explicate the seven-decade institutional changes and evolution of emergency management in China, resulting in analyses that often remain at the level of historical periodisation and characterisation, failing to elucidate the deep-seated logic of transformation. Secondly, China’s emergency management system has developed with considerable rapidity. Existing studies often reference dated cases (such as the SARS outbreak in 2003, the southern snow disaster in 2008, etc.) that represent only the state of emergency management at that specific juncture, failing to capture the significant impact and transformation precipitated by recent focal events (such as the COVID-19 outbreak in 2020) on China’s emergency management model. Thirdly, although existing research has explored the future development of emergency management in China, the proposed strategies are frequently rather generalised, lacking in practicality and operational applicability derived from theoretical analysis and identified practical dilemmas.

In view of this, this study endeavours to integrate Punctuated Equilibrium Theory (PET) with China’s emergency management practices by combining the long-term dynamic analysis framework of Punctuated Equilibrium Theory (PET) with the case study method. It seeks to explore the evolution logic of China’s emergency management system over more than 70 years since 1949, its future optimisation strategies, and to reveal its inherent discontinuous equilibrium characteristics. The aim is to furnish theoretical references and practical insights for the optimisation of global emergency management systems.

## 2 Research design and methodology

This study employs a qualitative, longitudinal case study design to analyze the evolution of China’s emergency management system from 1949 to the present. Our analysis is guided by an adapted Punctuated Equilibrium Theory (PET) framework. The methodological approach is detailed below.

### 2.1 Period delineation criteria

The four equilibrium periods identified in this study were delineated based on two primary criteria signalling a fundamental shift in the policy regime: (a) Major Institutional Reorganization, such as the creation of a new ministry-level agency (e.g., the Ministry of Emergency Management in 2018) that consolidates previously dispersed functions; and (b) Enactment of Foundational Legislation, such as the Emergency Response Law of the People’s Republic of China (2007), which establishes a new legal and operational framework for the entire system.

### 2.2 Data sources and collection

This research draws on a comprehensive analysis of primary and secondary sources. Primary sources include: (i) all national-level master and specific emergency plans released by the State Council; (ii) key legislation, including the Emergency Response Law (2007), the National Security Law (2015), and the Biosecurity Law (2020); (iii) official government white papers on disaster reduction and emergency management; and (iv) relevant sections of the National Five-Year Plans for Economic and Social Development. Secondary sources include a systematic review of academic articles in both Chinese (e.g., from CNKI) and English (e.g., from Web of Science) that analyze China’s emergency management history and specific disaster cases.

### 2.3 Focal event selection

The selection of focal events (e.g., the 2003 SARS outbreak) follows the criteria established in the policy studies literature (29). Events were included if they are consistently identified in both academic research [e.g., (30)] and official government retrospectives as critical junctures that exposed systemic failures, galvanized public attention, and directly led to a major policy agenda shift.

### 2.4 Analytical procedure: process-tracing

Our analytical procedure is a theory-informed qualitative process-tracing method. For each proposed policy punctuation, we systematically traced the causal pathway suggested by our adapted PET framework. This involved: (a) documenting the impact of a focal event or ideological shift; (b) analyzing changes in the policy image by tracking shifts in key terminology and problem framing within official documents, legal texts, and major speeches; and (c) analyzing changes in the policy venue by documenting institutional reforms, shifts in responsibility among government bodies, and the emergence of new coordination mechanisms. This method allows us to move beyond simple historical narration to provide a theoretically grounded explanation of the drivers and mechanisms of change.

## 3 Analytical framework: Punctuated Equilibrium Theory (PET) and its applicable amendments

### 3.1 Punctuated Equilibrium Theory (PET)

The concept of “discontinuous equilibrium” was introduced into the field of public administration by American scholars Frank Baumgartner and Bryan Jones in the 1990s (31). This perspective holds that the evolution of species is a combination of gradual quantitative change and punctuated qualitative change, where quantitative change is protracted and incremental, while qualitative change is abrupt and decisive, with the two occurring alternately rather than continuously (32). Punctuated Equilibrium Theory (PET), grounded in the methodology of paradigm research, explains the transmutation logic of policy system stability and mutation through the interaction of core variables such as policy venue, policy image, policy monopoly and its collapse, and focal events. It can be understood that public policy is formulated by the policy venue (which denotes the monopolistic or open subsystem composed of institutions, organisations, or groups possessing decisive authority over a specific issue or matter, (i.e., the institutional locus where policy authority is established) based on its policy image [the policy image refers to how the policy is understood and discussed by the public, the media, and social elites (31)]. In the early stages of policy implementation, the authoritative decision-making venue formulates public policy based on the prevailing policy image and maintains the closure and monopoly of the policy system for a certain duration through institutional barriers. During this period, when sudden focal events disrupt the extant equilibrium, if the current policy can respond effectively, a negative feedback mechanism will prompt gradual policy adjustments; conversely, if the policy fails, a positive feedback mechanism will impel a shift in the policy image, leading to the disintegration of the original policy monopoly. That is, policy opponents will establish a new decision-making venue by reconstructing the policy image, promoting a paradigm shift within the policy framework under bounded rationality and effecting a non-incremental punctuated evolution. New policies, upon being validated through practice, will enter a new stable cycle, and this cycle iterates to form a dual trajectory of policy evolution.

In this study, we refer to the underlying mechanism that drives the shift between policy stability and abrupt change as the ‘transmutation logic’ of the policy system. This logic is operationalised through the interaction of policy image, policy venue, and feedback mechanisms, as posited by PET.

### 3.2 The applicability of Punctuated Equilibrium Theory (PET) to the transformation of China’s emergency management system and its amendments

Given that Punctuated Equilibrium Theory (PET) originated from the practical context of Western public policy change, and recognising certain divergences between this context and China’s indigenous policy change environment, it is necessary to adapt the theory to the particularities of the transformation and evolution of China’s emergency management system to ensure the scientific validity of the research conclusions and the efficacy of the theory’s application. Firstly, the theoretical framework particularly accentuates the catalytic effect of sudden focal events. With the superimposition and resonance of traditional and non-traditional risk elements, contemporary Chinese society is prone to emergent focal events that breach norms, causing significant disruption to the operational environment and policy framework of the extant emergency management system. The abrupt institutional environmental changes triggered by such focal events align with the core assumption within the theoretical model that the evolutionary process is subject to punctuated change due to external shocks.

Secondly, from the perspective of the intrinsic attributes of the focal event, its non-linear characteristics such as suddenness, unpredictability, and irregularity are intrinsically congruent with the mutation mechanism explicated by Punctuated Equilibrium Theory (PET), thus underpinning the localised application of the theory.

**Figure 1:**
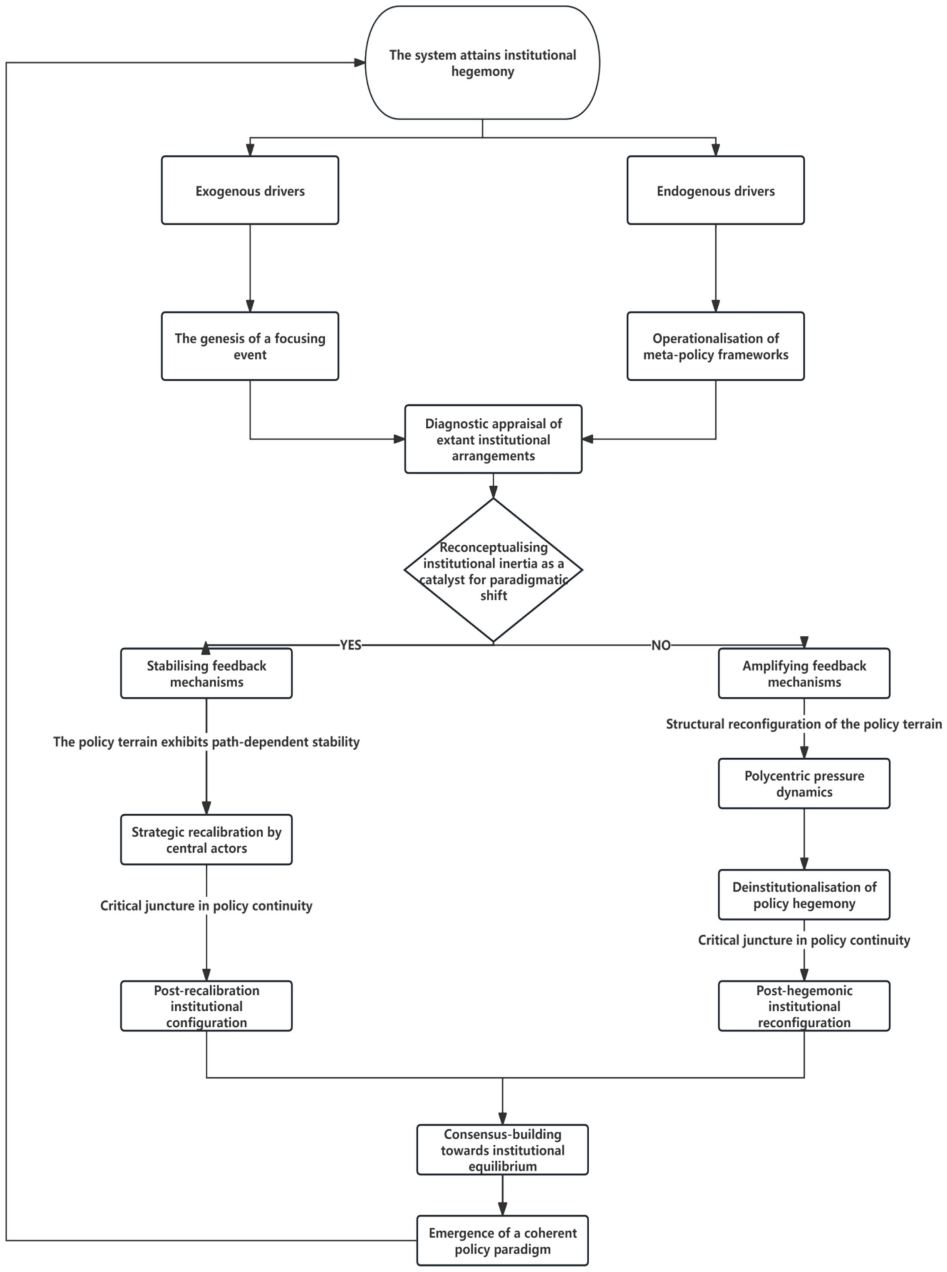
Revised framework for the evolution of China’s emergency management system based on Punctuated Equilibrium Theory (PET). The framework illustrates how exogenous drivers (focal events) and endogenous drivers (ideological shifts) interact to trigger policy punctuation. Policy image (how the issue is framed) and policy venue (institutional locus of authority) are key variables. Negative feedback maintains equilibrium through incremental adjustment, while positive feedback—amplified by punctuation—leads to monopoly collapse and paradigm shift.

Thirdly, within China’s unique political structure and decision-making mechanism, the evolution of the emergency management system is not solely triggered by focal events for punctuated adjustments but is also profoundly influenced by the cognitive iteration of decision-making subjects. Through the accumulation of experience derived from long-term governance practices, the decision-making stratum gradually discerns the efficacy boundaries of the extant system and consequently proactively implements gradual optimisation and internal restructuring. When major emergencies occur or governance concepts are innovated, the extant system faces multi-dimensional assessment: if the stabilising effect of institutional inertia exceeds the impetus for change, policy continuity will be maintained through incremental adjustments; conversely, when the driving force of innovation breaches the threshold of the old system, it triggers a shift in the policy paradigm and propels the emergency management system into a leapfrog development stage. Within this process, the new governance model gradually establishes a stable framework through institutional accumulation, constructing novel value consensus and policy legitimacy. This refined discontinuous equilibrium analytical framework (see Figure 1) can effectively map onto the historical evolutionary trajectory of China’s emergency management system. It is crucial to specify how this adaptation interacts with PET’s core causal logic. First, this modification extends PET’s core mechanism. In China’s state-centric system, an endogenous ideological shift, once formally codified by the Party-state, can itself function as a “top-down punctuation.” This decisive break can collapse the existing policy monopoly and mandate a new policy venue (e.g., the 2013 establishment of the National Security Commission following the proposal of the Holistic National Security Outlook), operating in parallel with the classic, bottom-up punctuation triggered by external focal events. Second, while an ideological shift may develop incrementally, its formal adoption as a national strategy or its codification in law represents a sharp, non-incremental break from the previous paradigm, fitting the definition of a punctuation.

This amendment addresses a long-standing debate in the comparative public policy literature concerning the extent to which policy process theories originating in Western democratic contexts can be applied to non-democratic regimes (33, 34). Research by van den Dool (16) demonstrates that while the core concepts of these theories retain diagnostic power in authoritarian contexts, their driving mechanisms indeed require recalibration due to differences in political institutions. Our ‘dual-driver’ model represents a concrete attempt at such recalibration. It acknowledges that in China’s state-centric system, an endogenous ideological shift formally codified by the ruling party (e.g., the ‘Holistic National Security Outlook’) can itself function as a ‘top-down punctuation,’ capable of collapsing the existing policy monopoly and mandating the establishment of a new policy venue. This operates in parallel with the classic, bottom-up punctuation mechanism triggered by external focal events. Furthermore, the findings by Chan (17) and Lam (18) that authoritarian regimes exhibit more intense policy punctuations provide corroborating evidence for our model: in the absence of diverse, independent information sources and robust societal challenges to the policy agenda, policy stability may be more pronounced, but when change does occur, its disruptive potential can be more severe. Therefore, our revised framework is not merely a summary of the Chinese experience but also a constructive extension and enrichment of PET from a comparative perspective.

To clarify the revised framework presented in Figure 1, it is essential to define its two core driving forces. Exogenous drivers refer to forces originating outside the established emergency management policy subsystem. These are primarily sudden, unpredictable focal events (such as the 2003 SARS outbreak or the 2020 COVID-19 pandemic) that generate significant social disruption and negative feedback, exposing the inadequacies of the current policy monopoly and forcing it onto the macro-political agenda. In contrast, endogenous drivers originate from within the policy subsystem itself, specifically from the strategic apex of the party-state. This encompasses proactive ideological shifts (e.g., the “Holistic National Security Outlook”) and top-down decisions for institutional restructuring (e.g., the creation of the Ministry of Emergency Management). These drivers represent a planned, rather than reactive, impetus for change. These two drivers are not mutually exclusive but interact dynamically. An exogenous shock can create a “policy window” that makes the system receptive to pre-existing endogenous ideas for reform. Conversely, a strong endogenous push for a new governance paradigm can enhance the system’s resilience, thereby shaping how it responds to future exogenous shocks. This dual-driver interaction forms the core of our adapted PET framework for analyzing China’s case.

## 4 Evolution process: the discontinuous equilibrium transformation of the emergency management system

**Figure 2:**
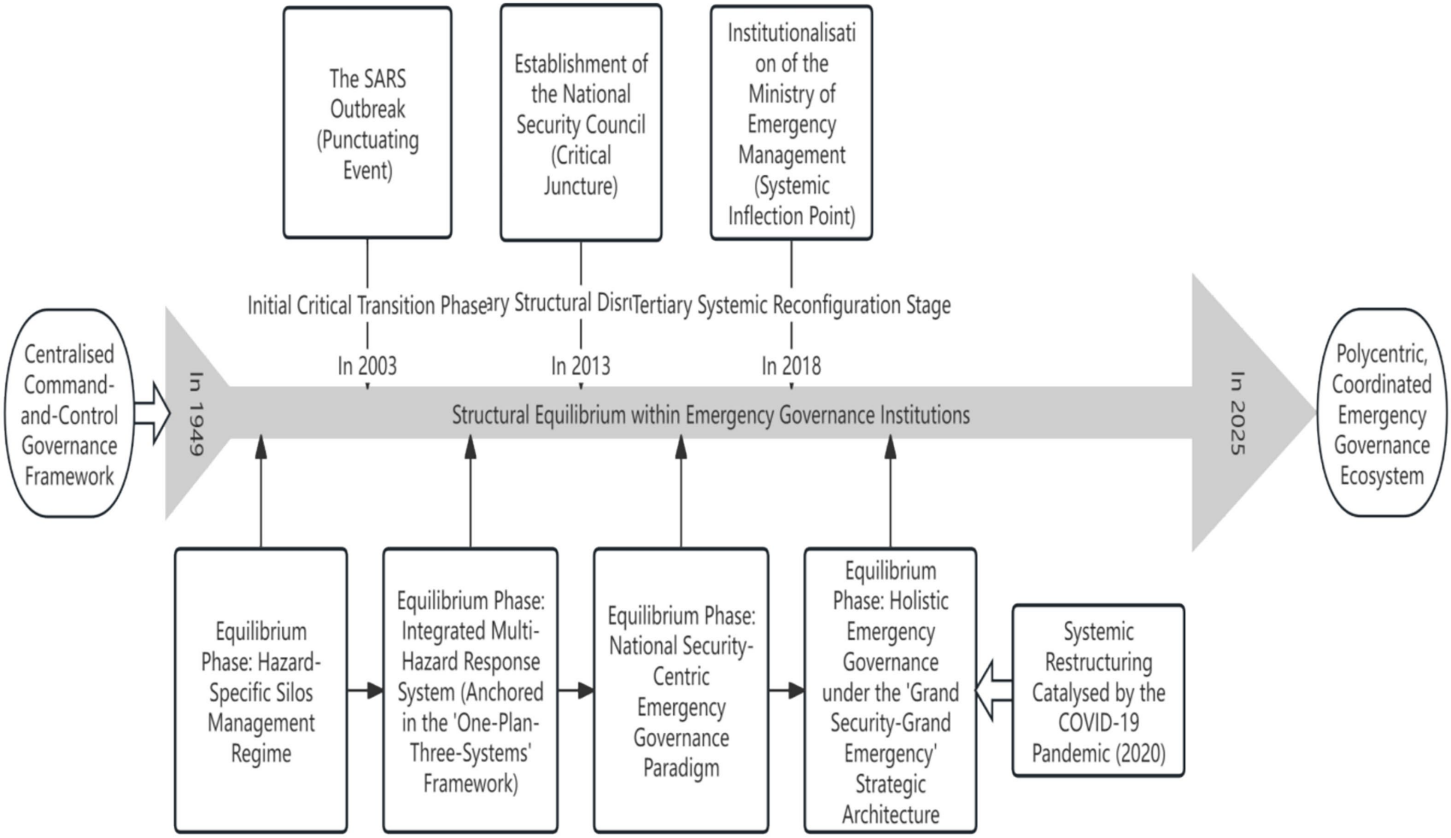
Evolution of China’s emergency management system based on discontinuous. Evolution of China’s emergency management system mapped onto the punctuated equilibrium model. Four equilibrium periods are separated by three major punctuations (SARS 2003, NSC 2013, MEM 2018) and one structural evolution (COVID-192020). The COVID-19 prompted institutional deepening within the existing “Big Security – Big Emergency” paradigm rather than a full punctuation.

From the perspective of Punctuated Equilibrium Theory (PET), the developmental trajectory of China’s emergency management system since 1949 can be delineated into four distinct stages: The equilibrium period of the disaster-specific management system primarily focused on single-hazard response (1949–2002), the equilibrium period of the multi-hazard integrated emergency management system centred on “One Plan, Three Systems” (2003–2012), the equilibrium period of the emergency management system under the holistic national security outlook (2013–2018), and the equilibrium period of the new era integrated emergency governance system under the “Big Security – Big Emergency” framework (2019–present). The discontinuous nodes were the SARS outbreak in 2003, the Wenchuan earthquake in 2008, the establishment of the National Security Commission in 2013, and the establishment of the Ministry of Emergency Management in 2018. Furthermore, the COVID-19 pandemic in 2020 also precipitated a degree of institutional restructuring within China’s emergency management (see Figure 2).

### 4.1 The equilibrium period of the disaster-specific management system based on single-hazard response (1949–2002)

#### 4.1.1 The nascent stage of the socialist system

For the 54 years from the founding of the People’s Republic of China in 1949 until the SARS outbreak in 2003, the concept of ‘emergency management’ was infrequently employed. As key reviews of the period suggest (26, 35), the relevant practices were better described as ‘disaster management’. At that time, China’s market economy and the risks associated with globalisation had not yet fully formed. The majority of public crises were dominated by natural disasters and epidemics. Influenced by the integrated management system of government, enterprises, and society, Party and government departments relied upon a robust political system for resource allocation. In handling emergencies, they typically adopted a vertical administrative command structure and implemented micro-interventions through campaign-style governance methods. This formed a single-hazard disaster management paradigm led exclusively by the government. Concepts such as “When one place is in trouble, help comes from all directions” constituted the mainstream ideology. Concerning fiscal management, the implementation of unified revenue and expenditure resulted in limited financial capacity for local governments, engendering an operational mechanism reliant upon central government fiscal support.

#### 4.1.2 From the era of reform and opening up to pre-SARS

Since the implementation of the reform and opening-up policy, latent risks have continuously accumulated. Disaster prevention efforts gradually extended beyond natural disasters to encompass work safety, public health, and social security (specialised regulations were formulated across these fields) within academia, the article “Crisis and Crisis Management” by Wei (35) is regarded as the inception of emergency management theory research in China. During this period, the emergency management model was characterised by categorised supervision, single-department handling, and *ad hoc* responses. The series of institutional arrangements formed under this policy image resulted in a phased policy monopoly. However, the extant policy system exhibited conspicuous limitations: insufficient systemic design, absence of cross-departmental coordination mechanisms, and weak comprehensive response capacity. When encountering major compound emergencies, these institutional shortcomings were fully revealed, creating the opportunity for subsequent punctuated changes (Table 1).

**Table 1:** Critical inflection points and their enduring consequences in post-1949 China.

Temporal phase	Focal event	Reference year	Principal consequence
The Era of Integrated Multi-Hazard Emergency Management Equilibrium: Centred on the “One Plan, Three Systems” Framework (2003–2012)	The Severe Acute Respiratory Syndrome (SARS) Epidemic	2003	Stimulated the systematic establishment of China’s emergency management system, marking the inaugural implementation of the foundational “One Plan, Three Systems” framework. The concepts of “emergency incident” and “emergency management” formally entered official policy discourse. The prominence of the social dimension of disasters eclipsed that of their natural dimension within academic and policy discourse. Precipitated the enactment of key emergency management legislation, notably the Emergency Response Law.
The Era of Emergency Management System Equilibrium under the Holistic National Security Concept (2013–2018)	Establishment of the National Security Commission	2013	Formulated the “Holistic Approach to National Security,” establishing a comprehensive governance framework for coordinating domestic and international emergency responses to all categories of hazards. Accelerated the promulgation of pivotal legislation, notably the National Security Law. Propelled the elevation of emergency management to the status of a national strategic priority through top-down institutional impetus.
The Era of Integrated Emergency Governance Equilibrium in the New Era: Within the “Comprehensive Security-Comprehensive Emergency Response” Framework (2019–Present)	Establishment of the Ministry of Emergency Management	2018	Centralised responsibilities for natural disaster prevention and mitigation, occupational safety regulation, and associated administrative functions. Promulgated regulatory instruments, including the Management Provisions for Standardisation in Emergency Response. Established an impetus for advancing integrated governance frameworks to higher operational tiers.
The Era of Integrated Emergency Governance Equilibrium in the New Era: Within the “Comprehensive Security-Comprehensive Emergency Response” Framework (2019–Present)	The Coronavirus Disease 2019 (COVID-19) Pandemic amidst Systemic Institutional Restructuring	2020	Institutionalisation of full-cycle governance mechanisms. Structural deconstruction of interdepartmental coordination barriers. Fundamental transformation of operational paradigms. Persistent challenges: Unresolved vertical-horizontal fragmentation, apparent legislative conflicts, and uneven resilience enhancement.

This extended equilibrium period illustrates the operation of a stable policy monopoly underpinned by a disaster-specific management paradigm. The policy image—centred on single-hazard response and campaign-style governance—remained largely unchallenged due to the absence of major exogenous shocks. Institutional inertia, reinforced by the planned economy and vertical command, sustained a negative feedback loop. However, the latent weaknesses—fragmentation, lack of coordination, and dependence on central fiscal support—laid the groundwork for a future punctuation. As PET posits, such equilibrium persists until a focal event amplifies positive feedback, leading to monopoly collapse and paradigm shift.

### 4.2 The first policy discontinuity: the SARS incident (2003)

The SARS outbreak in 2003 constituted the starting point for constructing China’s modern emergency management system. As a global outbreak of infectious disease, SARS initially received scant attention within China. It was not until mid-April when the epidemic proliferated nationally that it rapidly aroused profound concern among government agencies, the press, and the public due to its rapid transmission, unclear pathogenic mechanism, and the dissemination of social rumours. On 13 April 2003, the disease was formally incorporated into China’s list of legally notifiable infectious diseases. Four days later, following a dedicated study session by the Standing Committee of the Political Bureau of the Central Committee, the decision-making stratum fully acknowledged the severity of the epidemic and immediately initiated a series of emergency measures, including significant personnel adjustments. Under the coordinated response of the international community, global epidemic data stabilised by mid-July of that year, marking the conclusion of the acute crisis phase.

From the perspective of Punctuated Equilibrium Theory (PET), the SARS crisis triggered a fundamental shift in both the image and venue of China’s emergency management policy. Prior to 2003, the dominant policy image framed disaster management as a technical, single-hazard issue, addressed through fragmented, sector-specific responses; public health incidents were not yet conceptualised as requiring integrated emergency governance. The SARS outbreak shattered this image by revealing the systemic failures of a response model that lacked cross-departmental coordination, legal codification, and systematic preparedness. In its place emerged a new policy image that reframed public health emergencies as complex socio-technical events demanding comprehensive institutional reform. This transformation was accompanied by a decisive expansion of the policy venue: whereas decision-making authority had previously been dispersed across sectoral agencies such as the Ministry of Health and local governments with limited coordination mechanisms, the State Council now assumed a central coordinating role, and the subsequent construction of the “One Plan, Three Systems” framework institutionalised a new venue involving multiple ministries, legislative bodies, and expert advisory groups. The collapse of the old policy monopoly was driven by positive feedback—the failure of the existing system to contain SARS amplified public and elite attention, delegitimised the fragmented response model, and created irresistible political momentum for systemic reform. Thus, the crisis exposed the limits of the old equilibrium and accelerated the construction of a new institutional framework, marking the first major punctuation in the evolution of China’s emergency management system.

### 4.3 The equilibrium period of the multi-hazard integrated emergency management system centred on “one plan, three systems” (2003–2012)

During this period, China’s emergency management system achieved substantial progress. At the institutional level, the “state of emergency” was formally enshrined within the Constitution for the first time in 2004. The State Council issued framework guidelines for public emergency response plans, and in 2005, issued the National Master Plan, explicitly classifying public emergencies into natural disasters, industrial accidents, public health incidents, and social security incidents. Recognising the inadequacies exposed during the SARS response, an emergency plan system that is “horizontally comprehensive and vertically thorough” was progressively established. The promulgation of the Emergency Response Law of the People’s Republic of China marked the fundamental establishment of the legal framework for responding to public emergencies.

On the other hand, focal events during this period, such as the “5·12 Wenchuan Earthquake,” also contributed to the system’s evolution. It validated the efficacy of the response system but exposed shortcomings such as insufficient cross-departmental coordination and the lack of a legal basis for post-disaster reconstruction. Addressing these, The State Council promulgated the “Regulations on Post-Wenchuan Earthquake Recovery and Reconstruction” and revised relevant laws.

This phase demonstrates how a major punctuation (SARS) can catalyse the construction of a new policy monopoly—the “One Plan, Three Systems” framework. This represents a transformed policy image (from single-hazard to multi-hazard integrated management) and an expanded policy venue. The subsequent focal events, such as the Wenchuan earthquake, triggered negative feedback mechanisms that led to incremental refinements rather than a fundamental break, reinforcing the new equilibrium. However, the persistence of coordination failures indicated that the policy monopoly was weakening, setting the stage for the next punctuation.

### 4.4 The second policy discontinuity: the establishment of the National Security Commission (2013)

In November 2013, the Communist Party of China announced the formal establishment of the National Security Commission. Unlike discontinuities triggered by sudden focal events, this was a strategic choice made proactively by the central government—a quintessential example of an endogenous punctuation as defined in our adapted PET framework. The establishment of the Commission marked a decisive break from the previous policy paradigm, driven by an endogenous ideological shift: the formal proposal of the Holistic National Security Outlook. This new guiding doctrine fundamentally reconfigured the policy image of emergency management. Previously, emergency management was primarily framed as operational response to public emergencies, governed by the “One Plan, Three Systems” framework, with limited integration into broader national security strategy. Under the Holistic National Security Outlook, however, emergency management was elevated to a strategic national security concern, encompassing both traditional and non-traditional security threats—integrating public safety, biosecurity, and infrastructure protection into a unified strategic vision. This reframing necessitated a corresponding expansion of the policy venue. Whereas policy coordination had previously occurred within the State Council and sectoral ministries with limited involvement of Party-level strategic bodies, the National Security Commission—directly under the CPC Central Committee—became the apex coordination body, enabling cross-sectoral strategic planning and oversight beyond the administrative hierarchy. The trigger for this punctuation was not an exogenous crisis but an endogenous ideological shift—the Holistic National Security Outlook, formally adopted as a guiding doctrine, mandated institutional reconfiguration and collapsed the existing policy monopoly. This top-down punctuation provided a novel impetus for the evolution of the emergency management system, leading to another fundamental discontinuity in policy evolution.

### 4.5 The equilibrium period of the emergency management system under the holistic national security outlook (2013–2018)

Since 2012, public security incidents have exhibited diversified characteristics, forming a complex, intertwined risk network. In light of this, China commenced making decisions from the perspective of national strategy, signified by the proposal of the holistic national security outlook in 2014. Building upon this, the revision of the Emergency Response Law in 2017 explicitly defined responsibilities and established the principle of “unified leadership, comprehensive coordination, categorised management, and hierarchical responsibility.” In the same year, the report of the 19th National Congress explicitly stated that “we must resolutely fight the battle to prevent and defuse major risks,” signifying that China’s risk response had formally entered the stage of risk governance. However, flaws persisted, such as an imbalance in central-local authority and institutional vacuums in cross-departmental coordination, which set the stage for the third policy discontinuity.

This equilibrium period was shaped by an endogenous punctuation—the articulation of the Holistic National Security Outlook—which reconfigured the policy image from operational emergency management to a strategic national security concern. The policy venue expanded to include top-level party-state coordination. Despite these advances, the persistence of structural flaws reveals the limits of top-down institutional design without grassroots capacity adjustments. These unresolved tensions generated positive feedback pressures, paving the way for the 2018 reform.

### 4.6 The third policy discontinuity: the establishment of the Ministry of Emergency Management (2018)

In March 2018, the Ministry of Emergency Management of the People’s Republic of China was established, integrating responsibilities such as the prevention and control of natural disasters and the supervision of work safety. Subsequently, it promulgated the “Administrative Measures for Standardisation Work in Emergency Management,” further standardising the top-level design of standardisation within the field. The establishment of the Ministry of Emergency Management constituted not only an innovative attempt to meet the developmental demands of modern emergency management but also signalled the commencement of another systematic reform of China’s emergency management system.

From a PET perspective, this event represented a punctuation driven by positive feedback from long-standing institutional failures. Prior to 2018, the policy image of emergency management remained fragmented: responsibilities were dispersed across multiple ministries (e.g., Civil Affairs, Safety Supervision, Earthquake Administration), and despite the “One Plan, Three Systems” framework, recurring coordination deficits—especially during major disasters such as the 2008 Wenchuan earthquake—exposed the limits of a compartmentalised approach. The new policy image that emerged under the “Big Security – Big Emergency” framework envisioned a unified, all-hazards approach to emergency governance, integrating prevention, response, and recovery under a single ministerial command. This transformation was accompanied by a profound restructuring of the policy venue. Previously, responsibilities were scattered across 11 departments and 5 coordinating committees, leading to institutional fragmentation and inefficiency. The newly established Ministry of Emergency Management consolidated these disparate functions into a unified command structure, endowed with enhanced authority over resource allocation, information sharing, and cross-sectoral coordination. The trigger for this punctuation was the accumulation of negative feedback from recurrent coordination failures, which generated persistent political pressure for structural consolidation. This institutional redesign was both a response to these pressures and a proactive alignment with the Holistic National Security Outlook, thereby marking the third major punctuation in the evolution of China’s emergency management system.

### 4.7 The equilibrium period of the new era integrated emergency governance system under the “big security – big emergency” framework (2019–present)

During this period, China actively explored the construction of the “Big Security – Big Emergency” system, aiming to forge a new pattern of emergency management characterised by “Party leadership, government dominance, extensive participation of social forces, and market mechanisms.” This paper posits that the essence of this framework is systematic and integrated public safety governance. It should focus on strengthening the dual capacities of overall coordination and professional handling, aiming to form a risk governance community involving the collaborative participation of multiple entities.

The establishment of the Ministry of Emergency Management in 2018 marked a third punctuation, consolidating fragmented functions into a unified command structure and embodying the “Big Security – Big Emergency” policy image. This institutional redesign sought to overcome the coordination deficits of the previous equilibrium by expanding the policy venue to include cross-sectoral integration. However, as the COVID-19 pandemic revealed, the new framework remains incomplete. This ongoing equilibrium is therefore dynamic, with the potential for further punctuation as feedback from implementation accumulates.

### 4.8 Structural evolution rather than fundamental disruption: institutional restructuring achieved by the COVID-19 pandemic (2020)

As the most severe global public health crisis of the century thus far, the pandemic subjected China’s emergency management system to an unprecedented stress test. Its impact can be summarised as effecting a deep re-engineering of the institutional core within the “Big Security – Big Emergency” system, promoting the elastic expansion of the framework itself, yet without fundamentally breaching the foundational paradigm of the original system. Consequently, this paper categorises this change as “structural evolution” rather than “fundamental disruption.”

Applying the PET lens, the COVID-19 pandemic functioned as a powerful exogenous shock that triggered positive feedback, exposing critical institutional gaps and generating pressure for deepening reforms—yet it did not overturn the existing policy paradigm. Before the pandemic, the policy image of the “Big Security – Big Emergency” framework, while integrated in principle, remained unevenly institutionalised: gaps persisted in biosecurity legislation, digital governance integration, and grassroots resilience. The pandemic dramatically reframed and deepened this image, incorporating full-cycle governance, biosecurity legalisation, digital infrastructure integration, and systematic social participation as core components of emergency governance. In terms of the policy venue, the crisis catalysed significant expansion and institutionalisation. Whereas coordination had previously occurred through the Ministry of Emergency Management and sectoral agencies with limited cross-sectoral digital integration and weak public–private partnerships, the State Council Joint Prevention and Control Mechanism was elevated from a temporary entity to a permanent coordination platform. Digital platforms such as health codes integrated government and corporate actors (e.g., Tencent, Alibaba), creating a novel public–private governance venue. Grassroots communities and volunteers were more systematically embedded into the response network. These changes, however, unfolded within the existing “Big Security – Big Emergency” paradigm rather than replacing it; the foundational logic of unified command and integrated governance remained intact. Therefore, this event is best understood as structural evolution—a deepening and expansion of the existing framework—rather than a full punctuation that would entail a paradigm shift.

#### 4.8.1 Achieve vertical deepening: institutional anchoring for full-cycle governance

The outbreak and spread of the COVID-19 pandemic prompted a breakthrough in China’s emergency legislation. The newly enacted Biosecurity Law constructs a tripartite control system of “pathogen – laboratory – prevention, control and treatment,” formally incorporates public health security into the pillar of national security, and achieves the legal integration of biosecurity and major emergency response. Furthermore, this sudden public health event catalysed innovation in China’s emergency management mechanism, establishing a trigger standard for “peacetime-emergency conversion” and transforming “Big Emergency” from a concept into an operational institutional switch.

#### 4.8.2 Achieve horizontal integration: physical breakthroughs in cross-departmental collaboration

The pandemic impelled China’s emergency command system further towards materialisation. The State Council’s Joint Prevention and Control Mechanism was elevated from a temporary entity to a permanent coordination platform, granted legal authorisation to directly mobilise resources from departments such as health, transportation, and customs. Additionally, pertinent data barriers were progressively dismantled. The National Emergency Management Big Data Platform achieved three major breakthroughs: Firstly, during the public health emergency, a practical, crisis-driven interoperability measure was implemented to facilitate the sharing of specific, necessary data for epidemic control, operating under the broad authority granted by the state of emergency. This pragmatic approach, while effective, has subsequently sparked important scholarly and policy debates about the long-term balance between public health imperatives and the protection of personal information, as enshrined in laws like the Personal Information Protection Law; Secondly, it breached departmental data sovereignty through real-time comparison of inbound personnel data between the health system and the civil aviation reservation system; Thirdly, it facilitated an unprecedented level of real-time data collaboration between government and enterprises, for example, through the integration of health code systems developed by technology firms like Tencent and Alibaba into the national epidemic prevention framework, a public-private partnership that was crucial for mobility management and contact tracing (36).

These breakthroughs, while operationally effective during the acute phase of the pandemic, have raised profound questions about the long-term balance between public health imperatives and individual privacy. Critics have pointed to the risk of ‘function creep’, where exceptional measures become normalised and extended beyond their original purpose (36). The opacity of algorithmic decision-making in health code systems, combined with the absence of robust independent oversight mechanisms, has fuelled concerns that digital governance may entrench surveillance rather than enhance resilience (37, 38). Furthermore, the reliance on corporate actors such as Tencent and Alibaba for critical infrastructure raises unresolved questions about accountability, data ownership, and the boundaries between public authority and private power. These tensions suggest that the integration of digital technologies into emergency management is not merely a technical achievement but a contested political process.

#### 4.8.3 Achieve kernel reconfiguration: gene-level modification of operational logic

The pandemic drove a profound transformation in the dynamics of China’s emergency management. At the decision-making logic level, transitioning from passive response based on historical experience to proactive prevention grounded in scenario deduction; at the resource mobilisation level, shifting from subordinate reserves and layer-by-layer approval to intelligent allocation utilising national strategic reserves and market anti-fragility; at the level of social participation, evolving from passive administrative mobilisation of volunteer services towards certified professional forces actively embedded within the command chain.

#### 4.8.4 Unfinished deep waters of reform

While the pandemic precipitated a series of profound changes to China’s emergency management architecture, it ultimately failed to achieve a fundamental paradigm shift—what PET would classify as a full punctuation. Instead, it exposed and exacerbated several deep-seated structural tensions that remain unresolved, suggesting that the current equilibrium under the “Big Security – Big Emergency” framework remains provisional and contested.

Firstly, the contradiction between vertical control and horizontal coordination persists. Despite the centralising intent of the 2018 Ministry of Emergency Management reform, implementation at sub-national levels remains uneven and incomplete. For instance, the transition towards ‘vertical management’ of emergency functions at the provincial level has encountered bureaucratic resistance and remains subject to *ad hoc* administrative intervention by local governments, particularly at the county level. This ‘vertical–horizontal fragmentation’ undermines the unified command principle and perpetuates the very coordination deficits the reform was designed to overcome.

Secondly, legal conflicts have become increasingly explicit, revealing the limits of existing statutory frameworks under extreme conditions. A salient example is the unresolved tension between compulsory isolation measures mandated under Article 41 of the Law on the Prevention and Control of Infectious Diseases and the right to personal freedom guaranteed by the Civil Code. Such legislative ambiguities create operational uncertainty for frontline implementers and expose governance to legal challenge, indicating that the institutionalisation of emergency powers remains incomplete.

Thirdly, resilience-building remains spatially and socially unbalanced. While major metropolitan areas such as Beijing, Shanghai, Guangzhou, and Shenzhen have set ambitious planning goals—including the construction of ‘15-min emergency response circles’ (43)—a significant developmental gap persists between these core cities and emergency network coverage at the county and township levels. This unevenness not only reflects disparities in fiscal and administrative capacity but also raises fundamental questions about the equitable distribution of security as a public good.

These persistent challenges suggest that the current equilibrium, while more integrated than its predecessors, has not yet achieved the stability characteristic of a mature policy monopoly. The absence of an Emergency State Law to clearly delineate central–local authority allocation during crises, and the incomplete internalisation of digital governance platforms into national infrastructure (the ‘double inflection points’), indicate that further punctuation—whether triggered by endogenous reform or exogenous shock—remains a distinct possibility. To provide a concise comparative overview of how these critical junctures reconfigured the core PET variables, Table 2 summarizes the shifts in policy image, policy venue, and trigger mechanisms across the major punctuation events discussed above.

**Table 2:** PET variables across major punctuation events in China’s emergency management evolution.

Event	Old policy image	New policy image	Old policy venue	New/expanded venue	Trigger mechanism
SARS (2003)	Single-hazard disaster response	Multi-hazard integrated emergency management	Fragmented sectoral agencies	State Council-led “One Plan, Three Systems” framework	Positive feedback from exogenous shock
NSC Establishment (2013)	Operational emergency management	Strategic national security concern	State Council and ministries	National Security Commission (CPC-led)	Endogenous ideological shift (Holistic National Security Outlook)
MEM Establishment (2018)	Fragmented multi-ministry coordination	Unified “Big Security – Big Emergency” command	11 departments, 5 committees	Ministry of Emergency Management (consolidated)	Positive feedback from accumulated institutional failures
COVID-19 (2020)	“Big Security – Big Emergency” in principle	Full-cycle governance, biosecurity legalisation, digital integration	MEM and sectoral agencies	Joint Prevention Mechanism; public–private digital platforms	Positive feedback from exogenous shock (structural evolution)

## 5 Internal logic: non-linear evolution of the emergency management system

Since 1949, China’s emergency management system has been propelled by the dual forces of external focal events (bottom-up drive) and internal governance concept innovation (top-down design), jointly promoting the four-stage iteration of the system construction as follows:

### 5.1 Following the methodological approach outlined in section 2, which involved systematic analysis of key policy documents and legal frameworks, we observe that the policy image has undergone a transformation from itemised disaster management primarily based on single-hazard response to multi-hazard integrated emergency management centred on “one plan, three systems”—emergency management under the holistic national security outlook—integrated emergency governance in the new era under the “big security – big emergency” framework

This evolution is empirically evidenced by shifts in the core terminology and guiding frameworks enshrined in national policy. Firstly, the intertwining of focal emergencies and the governance concepts of the decision-making stratum has driven innovation within policy systems. Each reconfiguration of the decision-making subject’s cognitive framework precipitates adaptive changes in institutional design, and the emergency management models of each historical stage essentially constitute pragmatic projections of the dominant governance concepts. Taking the early PRC as an example: to ensure the stable operation of the agricultural production system and address frequent natural disasters, various departments and institutions were established, promoting “human wave tactics” and “collectivism” to manage specific sudden disasters. Post-reform and opening up, risk types became increasingly complex, and the governance focus gradually extended to work safety, public health, and social security. Nevertheless, prior to the SARS epidemic, China’s emergency management system still exhibited decentralised characteristics, its core logic remaining at the level of single-risk response.

With the continuous development of productive forces and the advancement of urbanisation, systemic risks manifested an exponential growth trajectory. The frequent occurrence of major emergencies persistently exposed the limitations of the traditional disaster-specific management model, compelling the emergency response system towards integrated transformation. With the normalisation of cross-border and complex disasters and the deepening of emergency response practices, the decision-making stratum commenced reconstructing the governance framework from the perspective of national strategic security, manifested by the establishment of the National Security Commission and the formation of the Ministry of Emergency Management. This top-level design innovation not only integrates traditional and non-traditional security affairs but also significantly enhances the efficiency of risk handling in complex situations, marking a formal shift in governance concepts towards modern emergency management under the guidance of the holistic national security outlook. Subsequently, domestic and international emergencies have occurred frequently, exhibiting multiple characteristics such as cross-border, complex, and diffused nature. Events such as the COVID-19 pandemic necessitate further optimisation and adjustment of the extant system by the Chinese government, further guiding enterprises, society, non-profit organisations, and other multiple entities to participate reasonably and orderly in the prevention, control, and governance of complex risks. Collectively, they constitute the new era of integrated emergency governance under the “Big Security – Big Emergency” framework.

### 5.2 The policy venue has transitioned from “unitary dominance and centralised management” to “multi-dimensional orderly collaborative governance”

The objective of building an emergency management system is to coordinate multiple entities to perform their respective tasks and functions within a complex system, thereby forming an overall synergy for an effective response to emergencies (39). However, prior to the SARS incident, the mainstream model of emergency management in China remained dominated by government departments as a single entity, with decision-making concentrated within official authorities, implementing a control and command-style disaster response model predicated on government, top-down, unitary dominance, and departmental responsibility. Limited public opinion was solicited during the formulation of emergency management policies at that time. In the 21st century, drawing upon international emergency practices, China’s institution-building has progressively trended towards strengthening risk prevention and source governance thinking, accelerating the institutional design of the public safety governance system. The landmark SARS outbreak not only verified the pivotal role of multi-party coordination mechanisms in enhancing emergency response efficiency but also revealed institutional shortcomings such as response lag and the absence of public opinion absorption mechanisms within local responses under the traditional control model. Objectively, it catalysed the systematic construction of China’s “One Plan, Three Systems” system. The concern of enterprises, news media, social organisations, and individual citizens regarding public safety generated substantial “grassroots pressure,” prompting the emergency policy formulation process to gradually incorporate social consultation mechanisms. Specifically, legislative bodies conducted public opinion solicitation concerning revisions to the Food Safety Law, and leveraged the role of higher education institutions and high-end think tanks in offering advice and suggestions. These measures further promoted the flattening and openness of China’s emergency governance structure. To a significant extent, China’s emergency governance achieved four major transformations: the strategic focus shifted from emergency response to risk prevention and control; the operational mode transitioned from passive response to active defence; the governance approach moved from administrative directives towards legal frameworks; and the responsible subjects evolved from departmental segmentation to comprehensive coordination, signifying a leap from traditional emergency management towards a modern governance system. However, due to the failure to fully overcome the inertia of campaign-style governance within the bureaucratic framework, problems persist such as weakened motivation and blurred responsibility at the grassroots executive level, manifested in the inflexibility of the emergency command system, the lack of operability of contingency plans, low resource allocation efficiency, and obstruction within the information sharing mechanism.

Building upon this foundation, following the establishment of the Ministry of Emergency Management, China systematically promoted the reorganisation and optimisation of cross-departmental functions in emergency management. By coordinating departments and localities to jointly handle public emergencies and integrating various emergency forces and material reserve systems, the organisational structure of comprehensive emergency management was significantly optimised, and the systematicness, integrity, and synergy of emergency governance were substantially enhanced. Presently, governments at all levels in China still bear the primary governance responsibility for public crises and constitute the authoritative subjects for the distribution of various emergency governance resources and values. Nevertheless, a comprehensive and dynamic governance network involving government departments, social organisations, grassroots communities, market entities, and international institutions has been preliminarily formed, thereby propelling China’s emergency management into the stage of “multi-party, orderly, and coordinated governance.”

Despite these advances towards multi-party governance, social participation in China’s emergency management remains structurally constrained. Non-state actors—including volunteers, community organisations, and private enterprises—are often relegated to a subordinate role, mobilised through administrative fiat rather than integrated as genuine co-governance partners with autonomous agency. Legal frameworks for volunteer involvement remain fragmented, and grassroots organisations frequently lack the capacity, resources, and legal standing to engage proactively in policy formulation or operational coordination. This asymmetrical partnership perpetuates a state-centric model in which societal actors are expected to comply rather than co-create. The persistence of campaign-style governance, as noted earlier, further reinforces top-down command logic, limiting the space for bottom-up innovation and local experimentation. These participation deficits not only constrain the synergistic potential of multi-actor governance but also raise normative questions about the democratic legitimacy of emergency decisions that profoundly affect citizens’ lives.

### 5.3 The dual driving effect of major focal events has become increasingly prominent

Major focal events are frequently sudden, highly destructive, and possess significant negative connotations. They not only propagate rapidly over short durations and large scales via technologies such as the internet and new media, leading to extensive attention and discourse by non-governmental organisations, news media, and the general public, but also readily trigger extreme public opinion and precipitate sudden mass incidents. Conversely, the more severe the damage caused by the emergency and the wider its reach, the higher the propensity for societal participation and the demand for policy adjustment become, compelling decision-making departments to accord close attention and respond through institutional innovation. In this manner, the occurrence of major focal events exerts a dual driving effect upon the transformation of China’s emergency management system that cannot be disregarded: while impelling relevant departments and institutions to implement policy and institutional improvements from the top down, it simultaneously prompts the public to seek enhancement of the current emergency system from the bottom up, thereby fostering a constructive social emergency culture.

Since 1949, various types of major focal events have, to varying degrees, prompted the transformation and evolution of China’s emergency management system. For instance, the COVID-19 pandemic in 2020 posed a severe test to the Ministry of Emergency Management, established in 2018. While China demonstrated the significant advantages of the leadership of the Communist Party of China and the socialist system with Chinese characteristics in national disaster relief, it also exposed deep-seated issues such as the limited initiative and capacity of local governments in responding to large-scale emergencies, the absence of comprehensive biosecurity legislation, the gap in emergency reserves of medical resources, and the need to enhance the public health early warning system (40). In view of this, relevant departments accelerated the introduction of pertinent laws and regulations, the research, development, and administration of novel vaccines, and refined the emergency prevention and control mechanism for COVID-19. Furthermore, the pandemic catalysed the development of China’s public health system. The construction of a tiered medical system based on medical alliances emerged as a new trend, and all medications listed in the COVID-19 diagnosis and treatment plan were incorporated into the national medical insurance directory. Collectively, these developments shaped a novel policy image at the level of emergency management decision-making in China.

## 6 Optimisation strategy: the enhancement and optimisation of the emergency management system

The following optimisation strategies are derived inductively from the historical analysis presented in Sections 4 and 5. They respond directly to the institutional failures identified in each equilibrium period—such as coordination gaps, legal ambiguities, and uneven grassroots capacity—and are informed by the theoretical logic of PET, particularly the dynamics of policy image and venue expansion.

### 6.1 Expand the policy venue for multi-party collaborative governance based on “big security – big emergency”

Under the overarching framework of “Big Security – Big Emergency,” emergency management has long transcended the traditional model of single disaster type and single department response. Its complexity and coupling present unprecedented demands upon the governance network. However, multi-party collaboration within current emergency management still confronts a profound “venue dilemma”: the policy space is fragmented by administrative levels, departmental barriers, and functional boundaries; the participation channels for multiple subjects are obstructed, rights and responsibilities are ambiguous, and information flow is impeded; social forces are often relegated to a subordinate position of “cooperation” rather than “co-governance,” making it difficult to fully unleash synergistic potential and resulting in institutional bottlenecks constraining overall emergency response capacity. To surmount this predicament, efforts must be directed towards strategically expanding and restructuring the policy venue, extending the institutional space for collaborative governance in both depth and breadth:

#### 6.1.1 Vertical integration: demolishing hierarchical barriers

It is imperative to transcend the simplistic “central-local” command chain and construct a flexible and contextualised dynamic adaptation mechanism for rights and responsibilities. Under special circumstances such as catastrophe response, more extensive on-site decision-making and resource allocation authority should be delegated to the grassroots level, while simultaneously strengthening the strategic support and baseline guarantee function of higher levels to form a “resilient structure” of top-down linkage and mutual reinforcement, avoiding the “power inversion” that diminishes synergy efficiency.

#### 6.1.2 Horizontal integration: bridging departmental divides

Building upon the achievements of the “Big Emergency” management system reform, deepen the regular functional integration under the concept of “all disaster types, Big Emergency.” It is necessary to move beyond the physical co-location (“co-working”) and concentrate on the standardisation and interoperability of the contingency plan system, information platforms, and command and dispatch protocols. Establish a departmental lead and rotation mechanism predicated on risk assessment to achieve a fundamental shift from “physical addition” to “chemical integration.”

#### 6.1.3 Outward absorption: activating societal momentum

Extend the policy venue into the broader social space, incorporating market organisations, professional institutions, community self-governing forces, and volunteers into institutionalised collaborative networks. The crux lies in establishing a “governance inclusion” mechanism: by setting clear access criteria, capability assessment systems, legal safeguards, and resource support (such as insurance, tax incentives), endowing them with a standardised action identity and sustainable participation pathway to achieve the effective integration and orderly embedding of social emergency resources.

### 6.2 Strengthen the scientific and forward-looking nature of the policy image based on “big security – big emergency”

Under the strategic framework of “Big Security – Big Emergency,” the core challenge of new era integrated emergency governance lies in transcending the fragmented model of passive response and constructing a policy image that is both scientifically rational and strategically far-sighted. This vision must confront the dynamism, coupling, and uncertainty inherent in complex system risks, achieving precise and future-oriented policy design through institutional innovation and technological empowerment:

#### 6.2.1 Deep quantification of risk perception

Relevant authorities should establish an intelligent risk assessment system of ‘all disaster types – whole process’. This is an ambitious goal, but international precedents exist. For example, Japan’s Disaster Management Information System integrates real-time data for multi-hazard monitoring, and the EU’s Copernicus Emergency Management Service provides geospatial information for disaster response. However, scaling these pilots to a national system faces formidable barriers. Technically, achieving interoperability across thousands of disparate local and departmental databases remains a monumental challenge. Legally, the tension between data sharing for risk assessment and the protections afforded by the Personal Information Protection Law requires careful navigation, potentially through specific provisions for emergency management. Politically, overcoming bureaucratic fragmentation and departmental ‘data sovereignty’ to create a truly integrated picture is perhaps the most significant hurdle. Future efforts must address these barriers concurrently with technological development.

#### 6.2.2 Model embedding for decision support

Develop a “context-response” intelligent simulation system and construct city-level emergency simulation sand tables based on digital twin technology. Introduce predictive tools such as infectious disease transmission models and disaster chain models during contingency plan formulation, and quantify the expected losses of different intervention strategies through Monte Carlo simulation to realise a paradigm shift from empirical decision-making to evidence-based decision-making.

#### 6.2.3 Stress planning for extreme scenarios

Establish a stress testing mechanism for “black swan” events and develop tiered response plans for low-probability high-impact scenarios such as climate tipping point breakdowns and critical infrastructure paralysis. For instance, simulate a compound disaster scenario of “extreme heat + grid failure + medical system strain” to test the limits of cross-departmental collaboration and compel redundant infrastructure design.

#### 6.2.4 Source interventions for governance resilience

Incorporate resilience indicators into the rigid constraints of territorial spatial planning. Examples include reserving “flexible land” for emergency shelters during urban renewal; adopting a distributed architecture design for lifeline projects to mitigate destruction impact; equipping community grids with micro-emergency supplies smart warehouses, etc. The aim is to reduce systemic collapse risk through front-end intervention in spatial governance.

### 6.3 Promoting bidirectional empowerment: dynamic coupling of top-level design and grassroots momentum in emergency governance

Under the “Big Security – Big Emergency” framework, the core tension of new era integrated emergency governance resides in how the proactive innovation momentum of the hierarchical system and the reverse pressure emanating from social forces can be transformed into governance synergy. The two appear oppositional but in fact constitute the dual engines driving institutional evolution. The key lies in constructing a “bidirectional empowerment” governance structure, achieving a creative transformation of top-down reform imperatives and bottom-up demands through institutionalised channels.

#### 6.3.1 Achieve proactive innovation in top-level design: structural empowerment

Authorities should translate novel concepts such as “full-cycle management” and “resilient governance” into legal norms and standard systems. For instance, revise the Emergency Response Law to clarify the legal status of the principle “prevention first, peacetime-emergency integration”; establish technical standards like the “Protocol for Responding to Catastrophe Scenarios,” mandating that contingency plans cover complex disaster chains and compelling departments to transform their thinking through legal responsibilities.

Relevant departments should establish a substantively operational “Big Emergency Committee,” endowing it with rights for cross-departmental resource allocation, plan review, and drill supervision. Implement a “function list + negative list” system. For example, vertically reduce command hierarchy to increase the direct access rate of provincial—municipal—field command posts to exceed 90%; horizontally establish a “departmental responsibility white zone” system, pre-assigning responsible entities for emerging risks such as AI security incidents, thereby eliminating response delays caused by functional vacuums.

#### 6.3.2 Promote institutionalised absorption of grassroots energy: responsive empowerment

Relevant departments should establish a closed-loop mechanism for event review and policy iteration. For example, launching ‘tripartite evaluations’ (government self-assessment, third-party institutions, public hearings) post-major events, converting public opinion hotspots into ‘emergency policy enhancement lists’ (e.g., promoting the revision of metro flood control standards following the 20 July Zhengzhou rainstorm). While social media data could, in principle, serve as one of many informational inputs to detect emerging public concerns, any such use must be governed by a robust framework that addresses the risks of censorship, algorithmic bias, and opacity. The priority should be on developing legitimate and transparent channels for public feedback rather than automated, algorithm-driven policy triggers. This ensures that responsiveness is achieved through accountable human judgment and democratic deliberation, not unaccountable technological systems.

#### 6.3.3 Create a bidirectional linkage system gear: dynamic adaptation mechanism

Authorities can create flexible spaces for policy experimentation. For instance, establish emergency policy sandbox zones, permitting localities to innovate beyond existing regulations under controlled conditions: representative potential examples could include Shenzhen piloting ‘pre-tax deduction of enterprise emergency reserve funds’; Chengdu exploring the ‘community emergency bond’ financing model.

Endeavour to construct a collaborative network for knowledge production and establish a risk knowledge community. For example, the government should proactively open disaster case libraries and simulation systems; universities can provide modelling support for complex systems; technology companies develop crowdsourced risk maps (e.g., Tencent’s “Emergency Warning Plugin Platform”), forming a multi-linked knowledge value-added cycle of “data-driven – academic verification – practice feedback.”

## 7 Conclusions and prospects

This study has applied the discontinuous equilibrium framework to analyse the evolution of China’s emergency management system since 1949, elucidating its phased characteristics and underlying dynamics. The analysis identifies a pattern of four equilibrium periods punctuated by three major discontinuities and one restructuring, following a non-linear logic of “gradual equilibrium maintenance – policy discontinuity – new equilibrium reconstruction.” The system’s development throughout this cyclical process demonstrates increasing complexity and institutionalisation.

The system’s transformation is driven by the interplay of internal ideological shifts and external focal events, which are deeply intertwined rather than sequentially distinct. This is reflected in the evolution of the policy image from single-hazard response to integrated governance under the “Big Security – Big Emergency” framework, and a shift in the policy venue towards multi-party coordination. Future optimisation efforts should focus on expanding collaborative governance, enhancing the scientific basis of policy, and fostering synergy between top-level design and grassroots initiatives.

It must be emphasised that the theoretical framework offers one plausible interpretation of a complex historical process, serving as a heuristic tool rather than positing a deterministic law. The findings contribute to a more nuanced understanding of policy evolution in China’s specific institutional context.

This study has several limitations. First, regarding theoretical and methodological constraints, as a conceptual analysis, it prioritises certain variables (e.g., focusing events) potentially at the expense of others, and the interpretive findings require future empirical validation. Second, concerning contextual specificity, the conclusions are deeply embedded in China’s socio-political context, which may limit their direct transferability to other governance models. Third, data limitations inherent in relying on secondary sources and official accounts may introduce interpretive biases and overlook micro-level implementation details. Finally, the analysis of the most recent ‘Big Security – Big Emergency’ framework is necessarily provisional due to its contemporary and evolving nature.

Fourth, this study’s focus on a single national case, while advantageous for in-depth analysis, inherently limits the generalisability of its findings. Our ‘dual-driver’ model is derived from an analysis of China’s specific political and institutional context. Although we have endeavoured to engage in a dialogue with the comparative PET literature, the extent to which this model is applicable to explaining the evolution of emergency management in other authoritarian or transitional states remains to be tested. Research by Joly (41), applying PET to the realm of international politics, and by Shafi (42), offering a comparative analysis of policy dynamics across multiple countries during a crisis, demonstrates the value and methodology of conducting cross-national comparative studies. Future research should draw upon such approaches. Through systematic cross-national comparisons, the theoretical model proposed in this study can be tested and refined, thereby enabling a deeper understanding of the commonalities and differences in the evolution of emergency management systems across various political contexts.

In light of these limitations, future research should employ detailed case studies and process-tracing methods to test the proposed causal mechanisms. Systematic comparative studies with other countries would also be invaluable for identifying unique and common patterns in emergency management evolution. Notwithstanding these limitations, this study provides a structured heuristic for understanding the non-linear evolution of emergency governance in China.

Importantly, this study does not seek to endorse a particular governance model but to offer a theoretically grounded analysis of its evolution. The persistent tensions identified throughout this analysis—between centralisation and flexibility, efficiency and privacy, state leadership and societal participation—highlight that China’s emergency management system remains an unfinished project, subject to ongoing contestation and adaptation. While the dual-driver model of exogenous focal events and endogenous ideological shifts provides a useful heuristic for understanding policy punctuations, it should not be read as a deterministic framework that precludes alternative trajectories. Future research should critically examine how these tensions are negotiated in practice, whether they generate new punctuations, and how China’s experience compares with other national contexts. The normative implications of emergency governance—particularly the trade-offs between security and liberty, speed and deliberation, state capacity and democratic accountability—deserve sustained scholarly attention beyond the confines of any single case.

## Data Availability

The original contributions presented in the study are included in the article/supplementary material, further inquiries can be directed to the corresponding author.
